# An integrative framework combining Mendelian randomization, single-cell profiling, and experimental validation identifies FTMT as a mitochondrial–immune regulator in non-small cell lung cancer

**DOI:** 10.1186/s12935-026-04387-z

**Published:** 2026-06-16

**Authors:** Shouyong Xiao, Siyun Wu, Xianfeng Shao, Ming Chao, Qiubo Huang, Chen Ke, Jiaping Chen, Guangjian Li, Lianhua Ye

**Affiliations:** 1 Department of Thoracic Surgery I, The Third Affiliated Hospital of Kunming Medical University / Yunnan Cancer Hospital / Peking University Cancer Hospital Yunnan , Kunming, 650118 Yunnan China; 2 Health Examination Center, The Third Affiliated Hospital of Kunming Medical University / Yunnan Cancer Hospital / Peking University Cancer Hospital Yunnan , Kunming, 650118 Yunnan China

**Keywords:** Non-small cell lung cancer, FTMT, Mitochondrial ferritin, MICB, NKG2D, Mendelian randomization, Single-cell RNA sequencing, Immunometabolism, Pharmacogenomics

## Abstract

**Background:**

Mitochondrial iron handling and immune surveillance are intertwined in tumor biology. Mitochondrial ferritin (FTMT) buffers redox-active iron in mitochondria, while MICB is an NKG2D ligand that can promote anti-tumor cytotoxicity when expressed on tumor cells. However, whether FTMT has a causal relationship with non-small cell lung cancer (NSCLC) risk—and how this might connect to MICB biology—remains uncertain.

**Methods:**

We applied a genetics–omics–validation workflow. We conducted two-sample Mendelian randomization (MR) and mediation MR using plasma proteomic pQTL summary statistics (UK Biobank Pharma Proteomics Project) and NSCLC GWAS summary statistics from FinnGen (R12). To contextualize results in the tumor microenvironment, we interrogated single-cell RNA-seq datasets via TISCH2 and explored pharmacogenomic associations using GDSC. Mechanistic plausibility was tested in A549 lung adenocarcinoma cells using FTMT overexpression followed by qPCR and Western blotting.

**Results:**

Genetically predicted higher FTMT levels were associated with reduced NSCLC risk (IVW OR per 1-SD increase ≈ 0.91). Mediation MR suggested that MICB-related signals accounted for a modest proportion of the FTMT effect. Single-cell analyses showed FTMT enrichment in malignant epithelial cells, whereas MICB was expressed across multiple compartments. In vitro, FTMT overexpression increased MICB mRNA and protein abundance.

**Conclusions:**

Integrating genetic evidence, tumor-context transcriptomics, and cell-based validation supports FTMT as a protective factor for NSCLC and points to a mitochondrial–immune axis involving MICB. Observed differences between blood-based genetic proxies and tumor-cell experiments are consistent with context-dependent regulation (e.g., circulating MICB can reflect shedding, whereas tumor-cell MICB reflects surface stress-ligand programs).

**Supplementary Information:**

The online version contains supplementary material available at 10.1186/s12935-026-04387-z.

## Background

Lung cancer remains a leading cause of cancer mortality worldwide, and NSCLC accounts for most cases. Despite progress in targeted therapy and immunotherapy, relapse and resistance remain common, motivating the search for mechanistically grounded biomarkers and actionable pathways [1, 2]. Iron metabolism has emerged as a key contributor to tumor behavior: iron is indispensable for proliferation and mitochondrial function, but iron mismanagement can promote oxidative stress and genomic instability [3].

FTMT is a mitochondrial iron-storage protein that sequesters iron and limits redox-active iron availability within mitochondria. Human FTMT was originally described as an intronless gene product targeted to mitochondria [4], and subsequent work has outlined its regulation and roles in physiological and pathological contexts [5].

Immune surveillance, particularly through the NKG2D receptor–ligand system, is crucial for recognizing stressed or transformed cells. MICB is a stress-inducible ligand that can engage NKG2D on NK and cytotoxic T cells, promoting immune-mediated clearance [6, 7]. Tumors can evade this axis by reducing ligand display or by proteolytic shedding of MICA/MICB, generating soluble ligands that may dampen NKG2D function and facilitate immune escape [7, 8]. Therefore, mechanisms that alter MICB abundance and form (surface vs. soluble) are highly relevant to tumor immunogenicity.

Here, we integrate MR, single-cell profiling, and in vitro experiments to test whether FTMT is causally linked to NSCLC and whether MICB-related biology may mediate part of this relationship.

## Methods

### Study design

**Fig. 1:**
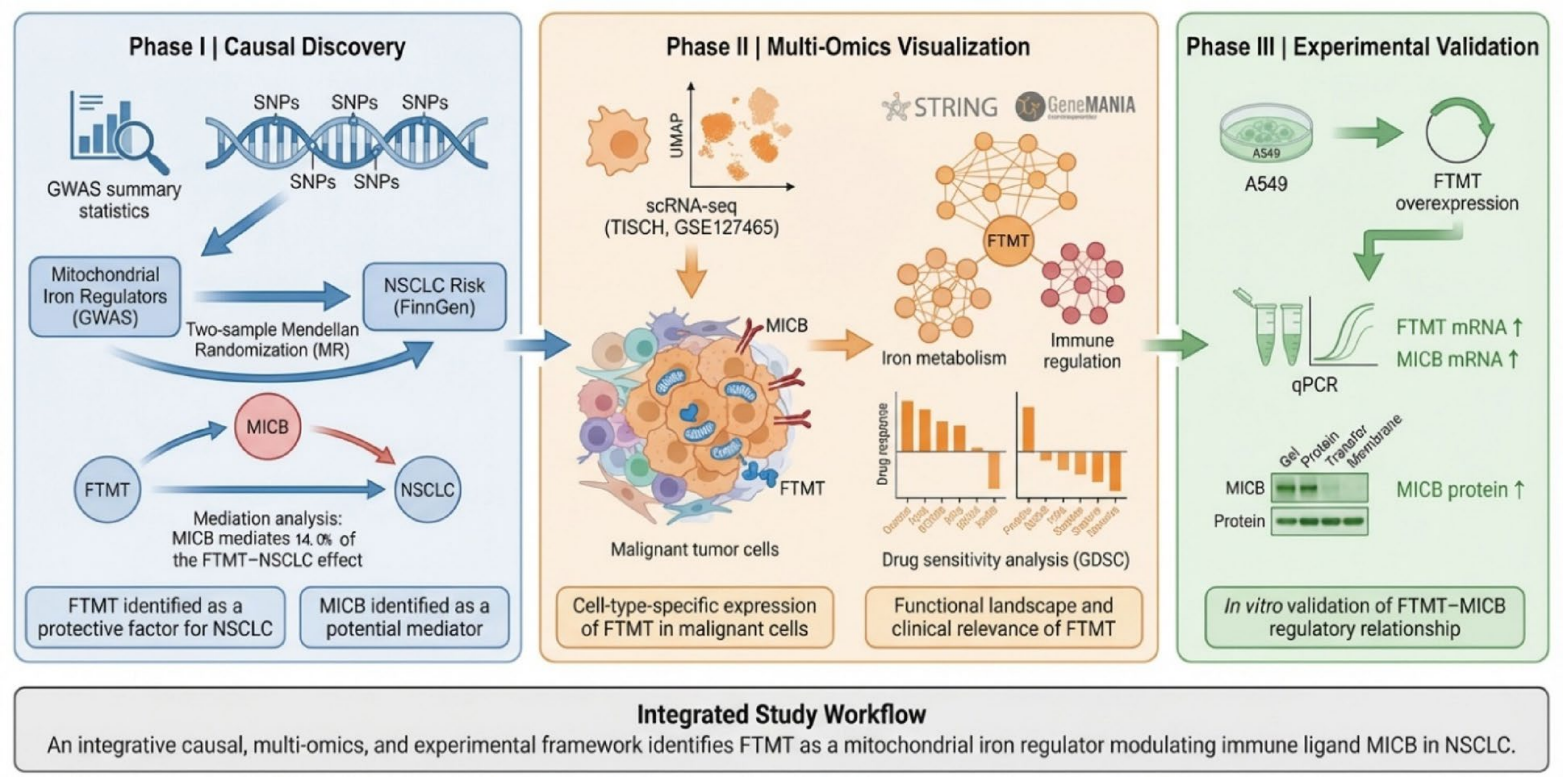
Comprehensive study workflow. Schematic flowchart illustrating the three-stage research design: (**1**) causal discovery via Mendelian randomization; (**2**) multi-omics mapping (single-cell, network, pharmacogenomics); and (**3**) in vitro experimental validation

The workflow is summarized in Fig. 1 and includes: (i) two-sample MR and mediation MR to evaluate FTMT–MICB–NSCLC relationships; (ii) tumor-context analyses using single-cell RNA-seq, co-expression and network resources, and pharmacogenomic correlations; and (iii) experimental validation using an FTMT-overexpression A549 model.

### Data sources

Exposure (protein) instruments were derived from publicly available plasma pQTL summary statistics from the UK Biobank Pharma Proteomics Project (UKB-PPP) [9]. NSCLC outcome summary statistics were obtained from FinnGen release R12 [10] using the endpoint definition in the FinnGen endpoint catalog [11]. Single-cell datasets were accessed via TISCH2 [12], including the NSCLC dataset built from GSE127465 [13, 14]. Bulk RNA-seq data for TCGA-LUAD were accessed through UCSC Xena [15, 16]. PPI analysis used STRING [17]. Drug response associations used GSCALite [18] integrating GDSC [19].

### Two-sample Mendelian randomization

Instrumental variables were selected at genome-wide significance (*P* < 5 × 10⁻⁸) and LD-clumped (r² < 0.001, 10,000 kb). Instrument strength was checked using F-statistics (F > 10). Primary causal estimates used inverse-variance weighted (IVW) MR, with sensitivity analyses using MR-Egger, weighted median, and weighted mode. Heterogeneity was assessed with Cochran’s Q; horizontal pleiotropy was evaluated using the MR-Egger intercept. MR-PRESSO was used to identify and correct for potential outliers [20]. Analyses followed established MR principles and reporting guidance [21–24].

### Mediation MR analysis

To estimate mediation by MICB-related signals, we decomposed the total FTMT→NSCLC effect into an indirect component via MICB and a direct component, using a product-of-coefficients approach consistent with network MR methods [25]. The mediation proportion was calculated using the product-of-coefficients method as (beta_a $\:\times\:$ beta_b) / beta_c, where beta_a represents the effect of exposure on the mediator, beta_b is the effect of mediator on the outcome, and beta_c is the total effect.

Single-cell transcriptomic and network analyses.

Single-cell transcriptomic visualizations (UMAP and feature plots) were directly exported from the TISCH2 web portal (dataset GSE127465), with differential expression internally assessed by the server via the Wilcoxon rank-sum test. Co-expression analyses were performed in TCGA-LUAD to identify genes correlated with FTMT. To further evaluate the downstream immunological consequences of the FTMT-MICB axis, we analyzed the Spearman correlation between MICB expression and key immune-effector molecules (GZMB, PRF1, and CD8A) in the TCGA-LUAD cohort (Supplementary Figure S1). Enriched modules were explored using STRING functional enrichment.

### Drug sensitivity association

Associations between FTMT expression and drug response (IC50) were explored using GSCALite’s standardized pipeline over GDSC data.

### Cell culture and experimental validation

A549 cells were cultured in DMEM/F12 with 10% FBS at 37 °C with 5% CO2. FTMT was overexpressed using the GV492 vector carrying the human FTMT coding sequence (NM_177478.2). At 48 h post-transfection, FTMT and MICB were quantified by qPCR (2^-ΔΔCt, GAPDH control) and by Western blotting (β-actin loading control). Data represent at least three independent experiments; two-tailed Student’s t-tests were used.

### Language editing statement

Language editing was assisted by an automated tool (ChatGPT, OpenAI) and used exclusively to improve grammar and clarity. All scientific content, analyses, and interpretations were generated and verified by the authors.

## Results

An integrative workflow links FTMT to MICB-mediated immune surveillance.

The overall study design is summarized in Fig. 1. We implemented an integrative workflow combining Mendelian randomization (MR), multi-omics contextualization, and in vitro validation to establish a coherent evidence chain linking FTMT to MICB-mediated immune surveillance in non-small cell lung cancer (NSCLC). This strategy enabled causal inference at the population level, spatial and functional interpretation within the tumor microenvironment, and mechanistic validation in cancer cells.

Mendelian randomization supports a protective role of FTMT in NSCLC.

**Fig. 2:**
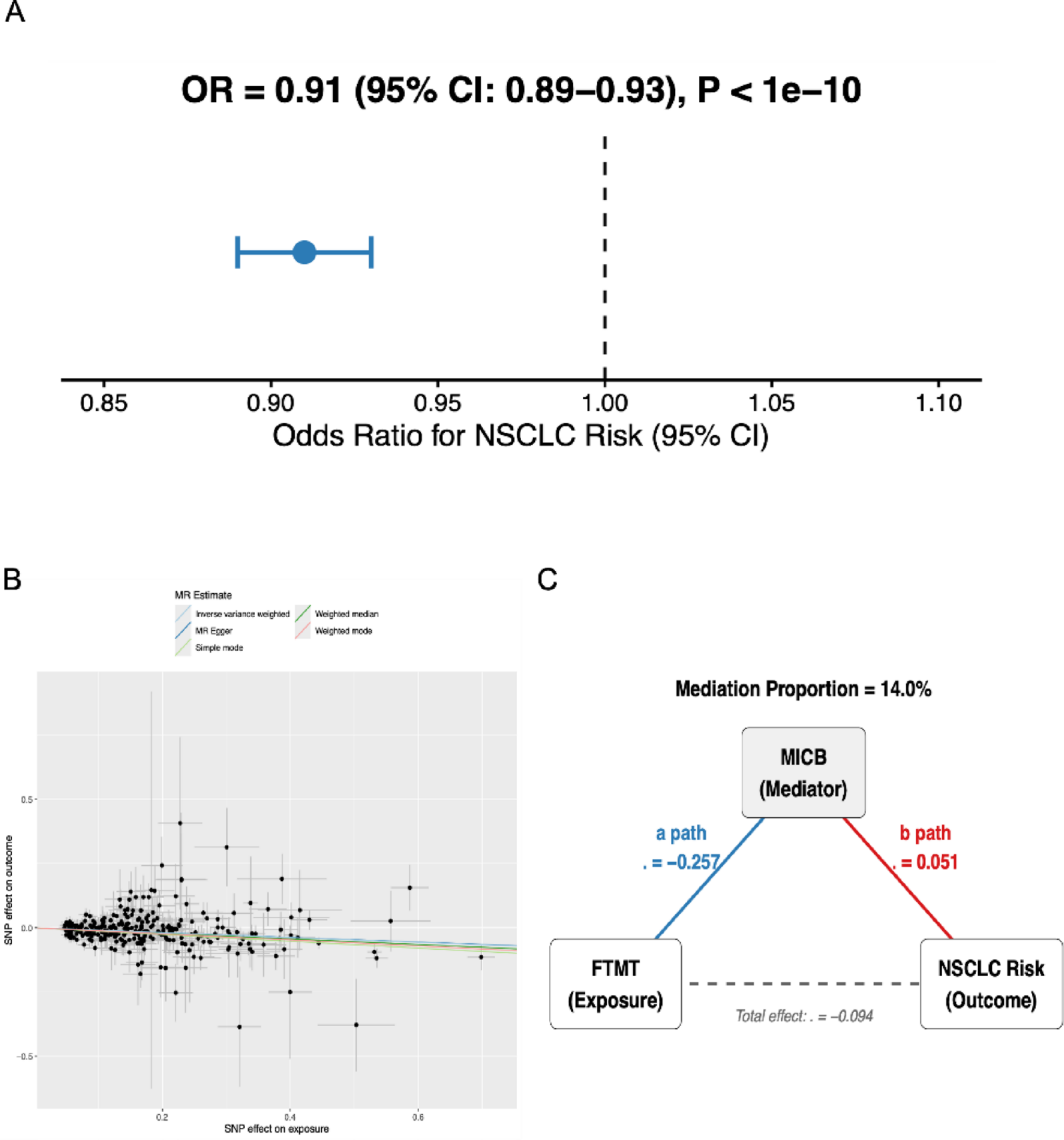
Causal and mediation effects of FTMT on NSCLC. (**A**) Primary MR effect estimates. (**B**) Scatter plot illustrating the consistency of SNP effects. (**C**) Mediation model showing MICB accounts for 14.0% of the protective effect

Two-sample MR analyses provided strong evidence supporting a protective role of FTMT in NSCLC. Higher genetically predicted FTMT expression levels were associated with a significantly reduced risk of NSCLC in the primary inverse-variance weighted (IVW) model (OR = 0.910, 95% CI 0.890–0.930, *P* = 1.15 × 10⁻¹⁶; n_snp = 276; Fig. 2A). The scatter plot visualized the robust relationship, showing consistent effect sizes across the instrumental variables (Fig. 2B). Directionally consistent effect estimates were observed across sensitivity analyses, including MR-Egger, weighted median, and weighted mode approaches. Comprehensive diagnostic testing, including forest plot, funnel plot, and leave-one-out analysis, did not indicate substantial horizontal pleiotropy or disproportionate influence from individual SNPs (Supplementary Figure S1).

Systematic MR screening across the candidate plasma proteome identified multiple proteins with genetically supported associations with NSCLC risk (Supplementary Table S1, Additional file 1). The strongest protective associations (OR < 1) were observed for BTN3A2 (OR = 0.907, 95% CI 0.886–0.928, *P* = 1.82 × 10⁻¹⁶; n_snp = 215), CCL19 (OR = 0.771, 95% CI 0.720–0.827, *P* = 1.97 × 10⁻¹³; n_snp = 61), AMBP (OR = 0.781, 95% CI 0.716–0.850, *P* = 1.45 × 10⁻⁸; n_snp = 57), DAPK2 (OR = 0.853, 95% CI 0.806–0.903, *P* = 3.72 × 10⁻⁸; n_snp = 58), and MPI (OR = 0.795, 95% CI 0.721–0.878, *P* = 5.12 × 10⁻⁶; n_snp = 32). In contrast, genetically predicted increases in BTN2A1, CDSN, HLA-E, PDIA5, and MICB/MICA were associated with increased NSCLC risk (overall OR range 1.05–1.22). Complete screening results are reported in Supplementary Table S1.

### MICB partially mediates the protective effect of FTMT on NSCLC

Given the immune-related signals identified in the MR screening, we evaluated MICB as a candidate mediator of the FTMT–NSCLC association. Genetically predicted FTMT expression was inversely associated with MICB levels (β = − 0.257, *P* = 9.93 × 10⁻²⁸), while MICB was positively associated with NSCLC risk (β = 0.051, *P* = 3.43 × 10⁻¹⁰). Based on the path coefficients (beta_a = -0.257, beta_b = 0.051, total effect beta_c = -0.094), the indirect effect was estimated at -0.013 [calculated as (-0.257 $\:\times\:$ 0.051)], accounting for 14.0% of the total protective effect (Fig. 2C). To further support the functional role of MICB in anti-tumor immunity, we analyzed its correlation with cytolytic effectors in the TCGA-LUAD cohort. MICB expression was significantly and positively correlated with GZMB, PRF1, and KLRK1 (Supplementary Figure S2), reinforcing the link between FTMT-induced MICB upregulation and enhanced immune surveillance.Full network MR and mediation outputs are provided in Supplementary Tables S2–S3 (Additional file 1).

### Single-cell and network analyses contextualize the FTMT–MICB axis

**Fig. 3:**
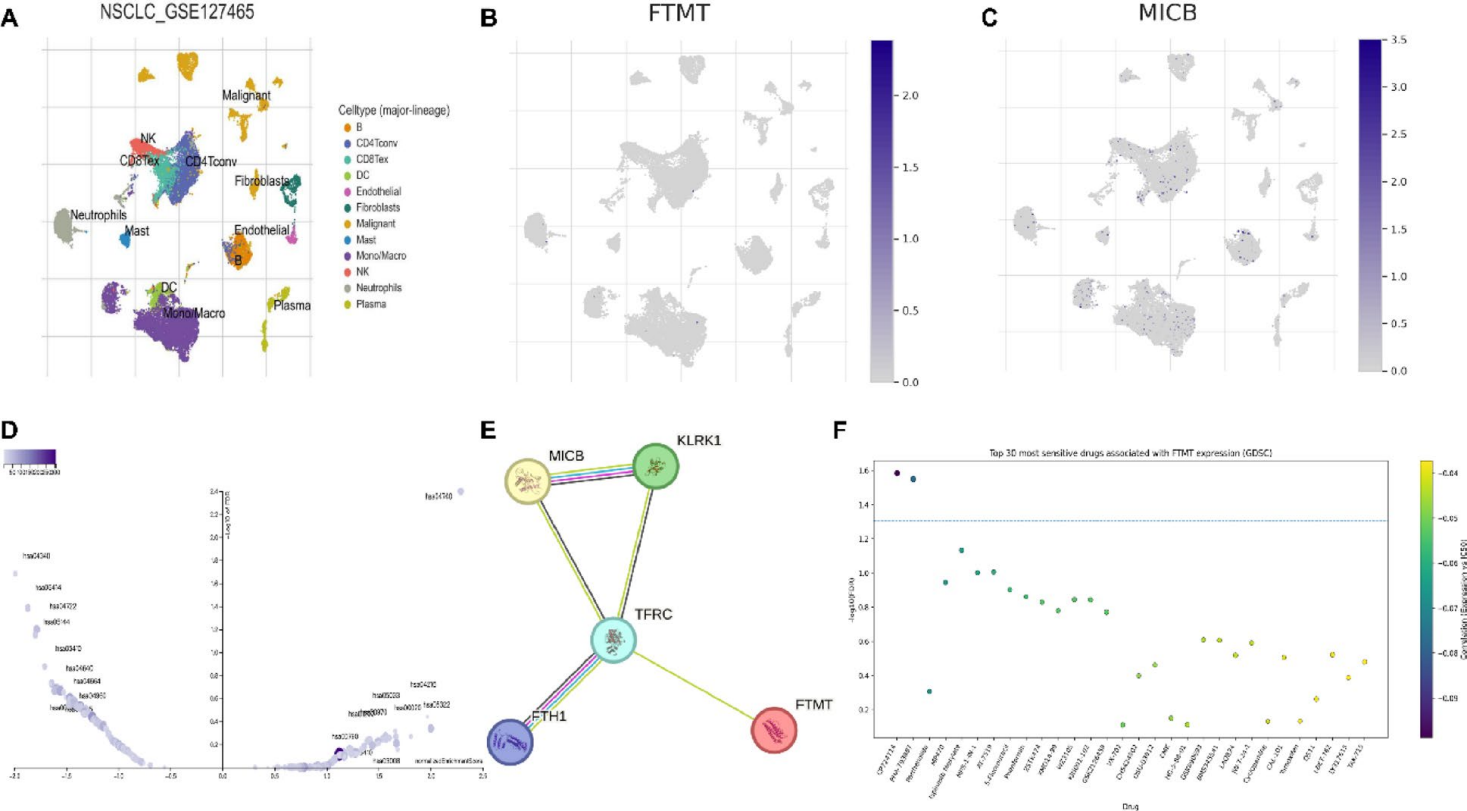
Multi-omics landscape of the FTMT–MICB axis. (**A**) UMAP visualization of the NSCLC tumor microenvironment. (**B**) Feature plot showing enriched expression of FTMT in malignant cells. (**C**) Feature plot showing the distribution of MICB expression. (**D**) Volcano plot of genome-wide correlations with FTMT. (**E**) STRING-derived PPI network highlighting functional connectivity of FTMT-associated genes. (**F**) Drug-sensitivity bubble plot (GDSC) showing correlations between FTMT expression and drug IC50 values

To investigate cellular context, we analyzed single-cell RNA sequencing data from the NSCLC_GSE127465 dataset. UMAP projections delineated major tumor microenvironment lineages (Fig. 3A). As illustrated in the UMAP feature plots obtained from the TISCH2 portal, FTMT expression was detectable and localized within the malignant epithelial cell populations. Due to the well-documented single-cell ‘dropout’ phenomenon affecting low-abundance transcripts, FTMT fell below the web server’s stringent automated pre-filtering thresholds for global adjusted P-value reporting; however, its spatial enrichment pattern remains visually evident. In contrast, MICB exhibited broader expression across multiple cell types (Fig. 3B–C), suggesting that FTMT may influence immune recognition through a stress-ligand program operating within tumor ecosystems.suggesting that FTMT may influence immune recognition through a stress-ligand program operating within tumor ecosystems.

Genome-wide co-expression analysis identified genes correlated with FTMT expression (Fig. 3D). Protein–protein interaction (PPI) analysis using STRING revealed functional connectivity between FTMT-associated modules related to iron and redox biology and immune-relevant nodes (Fig. 3E), providing network-level support for an immunometabolic link.

### FTMT expression correlates with drug sensitivity patterns

Pharmacogenomic analyses based on GDSC data indicated that FTMT expression was significantly associated with the IC50 values of multiple anticancer agents (Fig. 3F). These correlations suggest that FTMT may be linked to cellular stress vulnerability and treatment response, although these findings are exploratory and hypothesis-generating.

Experimental validation confirms FTMT upregulates MICB.

**Fig. 4:**
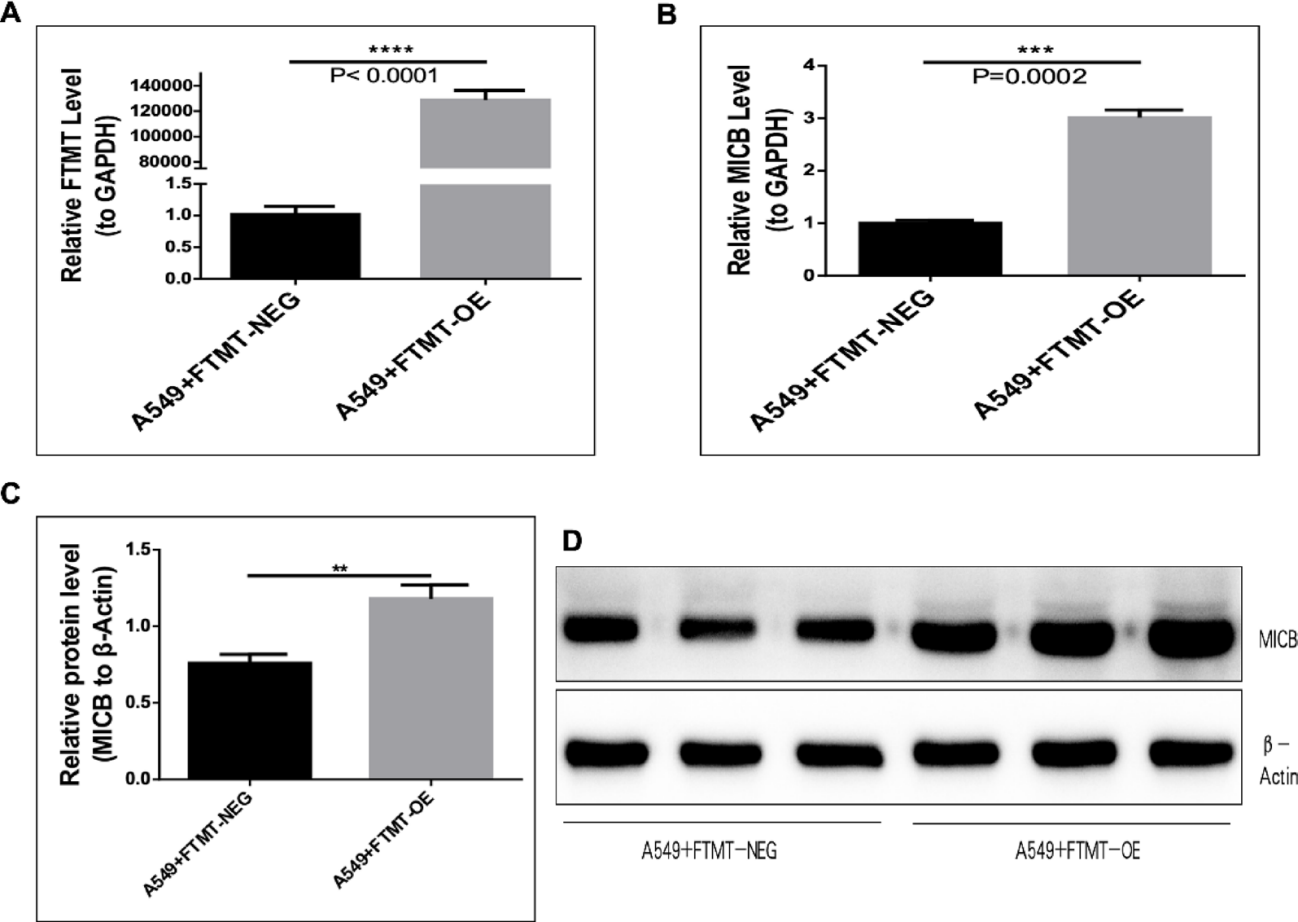
Experimental validation in A549 cells. (**A**) qPCR analysis of FTMT mRNA expression (Vector vs. OE). (**B**) qPCR analysis of MICB mRNA expression. (**C**) Representative Western blot images of MICB and β-actin. (**D**) Quantification of MICB protein levels. Data are mean ± SD. **P < 0.01, ****P < 0.0001

To experimentally validate the predicted FTMT–MICB relationship, we established an FTMT overexpression model in A549 lung adenocarcinoma cells (Fig. 4). Quantitative PCR confirmed robust upregulation of FTMT mRNA in the overexpression group compared with vector controls (Fig. 4A; *P* < 0.0001). FTMT overexpression significantly increased MICB mRNA levels (Fig. 4B; *P* < 0.01). Consistently, Western blot analysis and densitometric quantification demonstrated increased MICB protein abundance in FTMT-overexpressing cells (Fig. 4C–D; *P* < 0.01), supporting a positive regulatory relationship between FTMT and MICB at the cellular level.

## Discussion

By integrating Mendelian randomization, multi-omics profiling, and experimental validation, this study provides convergent evidence supporting FTMT as a protective factor in NSCLC and implicates MICB-mediated immune surveillance as a contributing mechanism. Our findings link mitochondrial iron homeostasis to tumor immune visibility across genetic, cellular, and experimental levels.

### FTMT and mitochondrial iron metabolism in cancer susceptibility

FTMT is a mitochondria-localized ferritin that sequesters iron and limits redox-active iron availability within mitochondria, thereby modulating oxidative stress and mitochondrial integrity [4, 5]. Dysregulated iron metabolism is a recognized hallmark of cancer, influencing reactive oxygen species generation, DNA damage, and metabolic reprogramming [3]. Compared with cytosolic ferritin subunits, FTMT specifically regulates mitochondrial iron pools, positioning it as a key modulator of mitochondrial stress responses. Emerging work has linked mitochondrial iron handling to ferroptosis and redox-dependent cell death pathways that are increasingly relevant to tumor biology and therapy response [26–28]. In this context, our MR results provide population-level causal support for a role of FTMT in NSCLC susceptibility.

### MICB links metabolic stress to immune surveillance

A central observation of this study is the involvement of MICB, a stress-inducible ligand for the NKG2D receptor that promotes cytotoxic responses by natural killer cells and cytotoxic T lymphocytes [6, 7]. Tumors frequently evade NKG2D-mediated immune surveillance through transcriptional repression or proteolytic shedding of MICA/MICB, generating soluble ligands that impair NKG2D signaling and facilitate immune escape [7, 8, 29]. Experimental strategies that inhibit MICB shedding have been shown to enhance NK cell–driven antitumor immunity, underscoring the functional relevance of MICB availability and form [8, 29].

Our mediation MR analyses indicate that MICB accounts for a modest but measurable proportion of the protective effect of FTMT on NSCLC risk. While MICB has been extensively studied in tumor immunology, upstream metabolic regulators of MICB expression remain incompletely characterized. Our findings suggest that mitochondrial iron buffering and stress responses may influence immune recognition by modulating MICB-related pathways, thereby linking immunometabolism to cancer susceptibility.

### Reconciling genetic associations with tumor-cell experiments

An apparent discrepancy in our study is the inverse association between genetically predicted circulating FTMT and MICB levels observed in MR analyses, contrasted with the positive regulation of MICB following FTMT overexpression in lung cancer cells. This difference likely reflects distinct biological layers captured by these approaches. Plasma pQTL-based MR reflects systemic, steady-state protein regulation and may partially capture soluble MICB derived from ligand shedding, which has been associated with immune suppression [7, 8, 29, 30]. In contrast, tumor-cell experiments primarily assess intracellular regulation and surface ligand expression under stress conditions.

Single-cell analyses provide important context by showing that FTMT expression is enriched in malignant epithelial cells, whereas MICB is broadly distributed across the tumor microenvironment. Together, these data support a context-dependent model in which FTMT induction within tumor cells enhances local stress-ligand programs, even when systemic circulating signals suggest an inverse relationship.

### Translational implications

Although primarily mechanistic, our findings have potential translational implications. Therapeutic strategies aimed at restoring NKG2D-mediated immune recognition, including inhibition of MICB shedding, are under active investigation [8, 29]. Our results suggest that mitochondrial iron metabolism, and FTMT in particular, may influence tumor immune visibility and potentially modulate responsiveness to immunotherapy. In addition, iron metabolism has been implicated in chemotherapy sensitivity and ferroptosis-based therapeutic strategies [27, 28, 31], raising the possibility that FTMT expression could inform treatment vulnerability in specific NSCLC contexts.

### Limitations and future directions

Several limitations should be acknowledged. First, a critical methodological limitation pertains to the nature of the instrumental variables used for FTMT. Upon strict genomic mapping, all 276 instruments were identified as trans-pQTLs, with no genome-wide significant cis-pQTLs located within the FTMT gene locus (Chromosome 5 ± 500 kb). While our rigorous sensitivity analyses (MR-Egger intercept *P* = 0.888) indicated an absence of directional pleiotropy, trans-pQTLs may act through complex upstream pathways. Consequently, our results should be interpreted as the effect of genetically predicted systemic FTMT regulation, and the complete absence of cis-instruments precluded a valid locus-specific colocalization analysis. Second, MR analyses rely on instrumental-variable assumptions and cannot fully exclude residual pleiotropy in complex immunometabolic pathways. Third, experimental validation was limited to a lung adenocarcinoma cell line, and additional NSCLC subtypes and patient-derived models are needed to assess generalizability. Fourth, although FTMT overexpression increased MICB expression, we did not directly assess immune cell–mediated cytotoxicity. Future studies incorporating NK cell or cytotoxic T cell co-culture assays will be essential to establish functional immune consequences.

In summary, this study integrates genetic inference, tumor-context transcriptomics, and experimental validation to support FTMT as a protective factor in NSCLC and to highlight a previously underexplored connection between mitochondrial iron homeostasis and immune stress signaling. Given the escalating public health burden of respiratory tract cancers, particularly in China where lung cancer incidence and mortality have surged significantly over the past three decades [32], elucidating novel protective mechanisms like the FTMT-MICB axis is of paramount clinical importance. These findings provide a framework for further investigation of immunometabolic regulation in lung cancer.

**Fig. 5:**
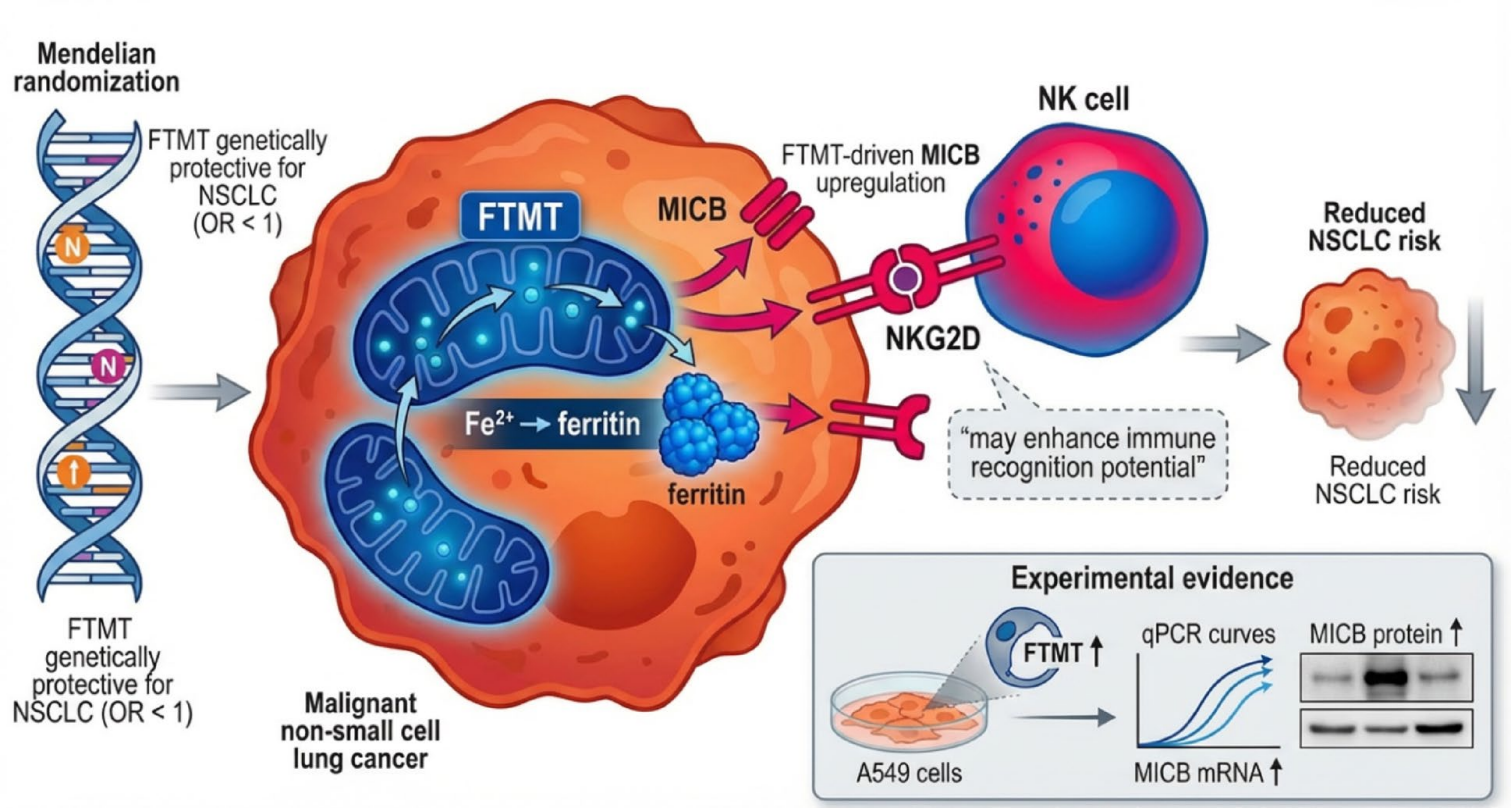
Proposed working model of FTMT-mediated protection against NSCLC. Mendelian randomization analyses support a protective role of FTMT in NSCLC at the population level. At the cellular level, FTMT regulates mitochondrial iron sequestration and ferritin formation, which under stress conditions may promote MICB upregulation in tumor cells. Increased MICB expression may enhance NK cell recognition through NKG2D engagement, contributing to immune surveillance and reduced NSCLC risk. Experimental validation in A549 cells supports FTMT-induced MICB upregulation. This model represents a hypothetical integration of genetic, mediation, and experimental evidence

## Supplementary Information

Supplementary Material 1: Figure S1. Robustness and sensitivity analyses for the MR study. (**A**) Forest plot of individual SNP effects. (**B**) Funnel plot to assess potential horizontal pleiotropy. (**C**) Leave-one-out sensitivity analysis.

Supplementary Material 2: Figure S2. Correlation between MICB and immune cytolytic markers in TCGA-NSCLC. Spearman correlation analysis demonstrates significant positive associations between MICB expression and (**A**) Granzyme B (GZMB), (**B**) Perforin 1 (PRF1), and (**C**) KLRK1. Data were derived from the TCGA-LUAD cohort.

## Data Availability

All data used in this study are publicly available. FinnGen Release 12 summary statistics for NSCLC (endpoint C3\_LUNG\_NONSMALL\_EXALLC) were downloaded from the FinnGen public data release portal (https://storage.googleapis.com/finngen-public-data-r12/summary_stats/release/finngen_R12_C3_LUNG_NONSMALL_EXALLC.gz). Plasma pQTL summary statistics were obtained from the UK Biobank Pharma Proteomics Project (UKB-PPP) resource. Single-cell transcriptomic data (NSCLC_GSE127465) were retrieved from GEO and analysed using TISCH2; protein–protein interaction analyses were performed using STRING; and drug-sensitivity correlations were assessed using GSCA and GDSC. [16–17] The full MR output tables underlying Fig. 2 (screening MR, protein-to-protein MR, and mediation MR results) are provided in Additional file 1 (Supplementary Tables S1–S3).
